# Reply: ‘Hand pattern indicates risk of prostate cancer’

**DOI:** 10.1038/bjc.2011.233

**Published:** 2011-07-26

**Authors:** A A Rahman, A Lophatananon, S Stewart-Brown, D Harriss, J Anderson, T Parker, D Easton, Z Kote-Jarai, R Pocock, D Dearnaley, M Guy, L O'Brien, R A Wilkinson, A L Hall, E Sawyer, E Page, J-F Liu, R A Eeles, K-R Muir, The UK Genetic Prostate Cancer Study Collaborators, British Association of Urological Surgeons' Section of Oncology

**Affiliations:** 1 Division of Epidemiology and Public Health, Queens Medical Centre, University of Nottingham, Nottingham NG7 2UH, UK; 2 Health Sciences Research Institute, Warwick Medical School, Warwick University, Coventry CV4 7AL, UK; 3 Nottingham Urology Centre, Nottingham University Hospital NHS Trust, Nottingham NG5 1PB, UK; 4 Royal Hallamshire Hospital, Glossop Road, Sheffield S10 2JF, UK; 5 School of Biomedical sciences, Queens Medical Centre, University of Nottingham, Nottingham NG7 2UH, UK; 6 CR-UK Genetic Epidemiology Unit, Strangeways Research Laboratories, Worts Causeway, Cambridge CB1 8RN, UK; 7 The Institute of Cancer Research, 15 Cotswold Road, Sutton, Surrey SM2 5NG, UK; 8 Department of Urology, Royal Devon and Exeter NHS Foundation Trust, Barrack Road, Exeter EX2 5DW, UK; 9 The Royal Marsden NHS Foundation Trust, Downs Road, Sutton SM2 5PT, UK; 10 Children's Brain Tumour Research, Division of Child Health, University of Nottingham, Queens Medical Centre, Nottingham NG7 2UH, UK

**Sir,**

We thank Cheema and Sharif (2011) for their interest in our paper. We agree that the strength of our study lies in its size, and therefore, we have been able to make reliable estimates of the association between digit ratio and prostate cancer risk. Epidemiological studies are able to estimate such relationships, but they are more limited in their ability to explain why such relationships occur, particularly in terms of the underlying biology as other possible explanations of any risks observed may exist. As such, the main purpose of our paper was to estimate the association between digit ratio and prostate cancer risk *per se* given the previously reported associations seen in a number of other cancers and indeed other conditions/traits. The explanation is that such risk may be driven by an underlying relationship with pre-natal hormone levels remains speculative, but would be in keeping with current thinking on the aetiology of prostate cancer. The evidence for a link between pre-natal hormones and digit ratio is suggested by McIntyre (2006) and Breedlove (2010).

In terms of any changes in the ratio of digit lengths with age or other factors, we are unaware of any data other than that of McIntyre *et al* (2005) and Trivers *et al* (2006) that suggest that it is generally longitudinally stable, and given that further collection of longer-term information on this would require much time and effort, we remain happy to present our main finding on the association to encourage other groups to study whether such a relationship is seen in other studies, given the interest in this externally available trait.

If our observations are replicated, the finding may further stimulate useful insights into the biology of prostate cancer risk and/or have clinical utility.
